# Alternative cancer clinics’ use of Google listings and reviews to mislead potential patients

**DOI:** 10.1038/s44276-024-00071-9

**Published:** 2024-08-06

**Authors:** Marco Zenone, Jeremy Snyder, May van Schalkwyk, Jean-Christophe Bélisle-Pipon, Greg Hartwell, Timothy Caulfield, Nason Maani

**Affiliations:** 1 Faculty of Public Health and Policy, London School of Hygiene and Tropical Medicine, Keppel St, London, UK; 2 Faculty of Health Sciences, Simon Fraser University, Blusson Hall, 8888 University Drive, Burnaby, BC Canada; 3 Health Law Institute, Faculty of Law, University of Alberta, Edmonton, AB Canada; 4 Global Health Policy Unit, The University of Edinburgh, Edinburgh, UK

## Abstract

**Background:**

Alternative cancer clinics, who provide treatment associated with earlier time to death, actively seek to create favorable views of their services online. An unexplored means where alternative cancer clinics can shape their appeal is their Google search results.

**Methods:**

We retrieved the Google listing and Google reviews of 47 prominent alternative cancer clinics on August 22, 2022. We then conducted a content analysis to assess the information cancer patients are faced with online.

**Results:**

Google listings of alternative treatment providers rarely declared the clinic was an alternative clinic versus a conventional primary cancer treatment provider (12.8% declared; 83.0% undeclared). The clinics were highly rated (median, 4.5 stars of 5). Reasons for positive reviews included treatment quality (*n* = 519), care (*n* = 420), and outcomes (*n* = 316). 288 reviews presented the clinics to cure or improve cancer. Negative reviews presented alternative clinics to financially exploit patients with ineffective treatment (*n* = 98), worsen patients’ condition (*n* = 72), provide poor care (*n* = 41), and misrepresent outcomes (*n* = 23).

**Conclusions:**

The favorable Google listing and reviews of alternative clinics contribute to harmful online ecosystems. Reviews provide compelling narratives but are an ineffective indicator of treatment outcomes. Google lacks safeguards for truthful reviews and should not be used for medical decision-making.

## Background

Alternative cancer clinics offer unproven, disproven, and questionable treatments with worse clinical outcomes than evidence-based cancer services [1, 2]. This includes unproven treatments such as Gerson therapy, among others. Frequently, the persons receiving treatment have a late-stage or terminal prognosis, have exhausted evidence-based medical options and thus are in search of hope of a cure or extending life [3]. In other cases, persons with cancer may forgo evidence-based medical opportunities [4] in favor of ‘natural’ or ‘holistic’ options for various reasons, such as misinformed perceptions of chemotherapy and radiation harm. 40% of Americans believe alternative treatment can cure cancer [5].

Despite worse outcomes, thousands of patients receive cancer services yearly from alternative cancer providers. Clinics actively seek to attract patients, including through social media and paid advertisements. Facebook support groups, created to help people and their families cope with a cancer diagnosis, are targeted by alternative cancer provider marketing [6]. Most alternative cancer clinics have active social media accounts [7] on Facebook, Instagram, YouTube, and Twitter, and now, several are expanding to TikTok. Alternative cancer clinics use paid Facebook and Instagram advertisements to appeal to potential patients [8]. Using advertisements, clinics present themselves as a legitimate medical option for cancer treatment despite questionable credentials and approaches [9, 10]. Clinics administering treatment rely heavily upon the use of apparent patient testimonials, that have not been verified by an independent source, to support their legitimacy.

Alternative cancer clinics face several challenges in their attempts to attract potential patients. Regulators at different branches of government have attempted to regulate predatory alternative cancer treatment marketing [11–13]. Some such clinics have received negative news coverage that asserts they offer ‘false hope’ and ‘misleading’ treatments to persons in desperate situations [14, 15]. Dedicated blogs by qualified medical professionals warn against their treatments [16, 17]. Increasing research activities and attention to alternative cancer treatment outcomes put the spotlight on the questionable practices of many such clinics [1, 2]. Certain alternative cancer clinics are subject to lawsuits from prior patients [18, 19]. In some cases, the families of persons who tried alternative treatment, and died, warn others of the risks [20]. Conventional cancer information authorities warn against alternative cancer treatment services [10, 21].

In response, clinics actively attempt to defend themselves on social media and their websites to shape and maintain their reputations. For example, Chipsa Hospital, an alternative cancer clinic in Tijuana, Mexico, has a web page titled: “Chipsa Hospital is No Scam See Why [22].” In rare cases, alternative cancer clinics threaten libel and legal proceedings against critics [23].

An unexplored means where alternative cancer clinics can shape their brand is their portrayal across web search results. Google listings, which provide a summary of a business, location, or person, also provide descriptive information on the purpose of a business. This information is often the first point of contact by a Google search user Googling the name of a business. For example, if prospective cancer patients search ‘clinic xyz’, they will see a clinic description. Businesses control how they identify themselves and appear to users [24]. For an alternative cancer clinic, whose services are unsupported by authoritative medical bodies, this can lead to opportunities to self-declare as a qualified organization for primary cancer treatment. A prospective patient seeing such information may as a result have a distorted impression of the clinic.

Additionally, search results contain Google reviews, where previous clients of a business can post a 1-to-5-star rating of their services and a text explanation outlining their rating rationale. For alternative cancer clinics, reviews are an important indicator to prospective patients about the quality of services and experiences of former clients. Online reviews influence decision-making for non-medical and medical businesses [25–27] and research has consistently shown that online reviews, particularly for professionals, can be very persuasive [28]. Unfortunately, there is a high potential for fraudulent activity in Google review ratings, where businesses rate themselves or hire an outside business to inflate their review score [29, 30]. Misleading testimonials represent a key concern for cancer patients using Google reviews as an indicator of treatment quality and efficacy. In addition, studies have found that online reviews are a poor predictor of objectively assessed quality care [31].

The information environment provided by Google listings and Google reviews may contribute in part to misleading potential cancer patients about alternative cancer clinic qualifications, reputation, and treatment efficacy. Understanding Google listing and review content is necessary to assess the information cancer patients are faced with when seeking care online. Therefore, this study seeks to answer: (1) how alternative cancer providers’ expertise and qualifications are portrayed in their Google listings; (2) how clinics are rated across their Google reviews; (3) for what reasons and outcomes Google reviews are rated positive (score 4 or 5) or negative (score 1 or 2); (4) if reviews contain an action statement recommendation to receive treatment from the provider; (5) who is making the reviews and in what situations; and (6) if reviews contain evidence of reputational management. The results can inform health policy to deter cancer patients from receiving unproven care and support advertising regulators to understand an unrecognized form of unproven medical advertising.

## Methods

The present study followed the standards for reporting qualitative research (SRQR) guidelines (a checklist is provided in SI1) [32]. The first step of our study was to identify the alternative cancer clinics for which we would collect their Google listing and reviews. Our study did not aim to collect the full spectrum of alternative cancer clinics which exist worldwide, which is likely unfeasible, but to identify prominent clinics. We sought to identify clinics which primarily offered as a key service, alternative cancer treatment. We retrieved a list of 47 alternative cancer providers identified in a previous study investigating how alternative cancer clinics market their services across paid Facebook and Instagram advertisements [8]. The study retrieved their list from a patient directory of alternative cancer clinic options (HealNavigator.com) and a study that investigated treatment destinations named where prospective cancer patients fundraised for alternative cancer treatment [3]. Taken together, our alternative cancer clinic identification strategy retrieves clinics actively courting patients online and with a vested interest in positive online portrayals.

We retrieved the Google listing and Google reviews of the 47 clinics on August 22, 2022. To collect the advertisements, MZ found the unique Google search URL of each clinic and created a Data Miner scraper [33] to collect listing information (name, description, location) and their Google review information (overall rating, number of reviews, each rating text content and score). This led to the retrieval of 1444 Google reviews. We assessed each review for inclusion by determining whether the Google review pertained to cancer treatment, as some clinics offered more than cancer services. However, although there are 1444 Google reviews identified, only 1046 contain text in their reviews. Many reviews give a rating (between 1 and 5 stars), sometimes without text. Therefore, our ability to exclude potentially non-relevant reviews is a limitation. We mitigated this by ensuring the alternative cancer clinics in our sample were either (1) known for their cancer services, (2) only provided cancer services, or (3) offered cancer services as a specialty or key component of their clinic. We included 1136 reviews, of which 738 contained review text.

MZ reviewed all data and developed a content analysis framework [34] to answer the research questions. Content analysis has been used in previous research investigating Google reviews [35]. After drafting the code frame, all other authors reviewed and provided feedback. The authors then agreed upon a final frame. The final coding frame is provided in Table 1. After code frame approval, MZ independently coded the data. JS randomly selected and audited 20% of all code applications on Google reviews with text (total, 738; audit total, 148). JS also audited codes labeled ‘sensitive in nature’ and thus required a full audit of each applied code. Sensitive codes included: financial exploitation; misrepresenting the impact of treatment on cancer; and improvement – cancer cured, improving, or in remission due to received treatment. Upon completion of the audit, JS and MZ received high agreement on coding and resolved all disagreements with discussion. Clinics and their associative code applications were anonymized after the study was completed.

**Table 1:** Coding frame overview: research question, data source, and code(s).

Research Question	Data Source	Code	Sub-code(s)
RQ1	Google listing	Listing description declares clinic is:	(a) alternative provider; (b) hospital, or medical and cancer clinic; (c) blank or unspecified.
RQ2	Google reviews	Total rating scores (0–5) average	Not applicable.
RQ3	Google reviews	Negative review reasons	(a) quality of facility; (b) quality of service provision (non-efficacy); (c) financial exploitation; (d) misrepresenting impact of treatment on cancer; (e) difficulty accessing treatment; (f) time away from loved ones; (g) treatment worsened patient conditions or cancer progressed; and (h) patient died.
		Positive review reasons	(a) quality of facility; (b) quality of service provision (non-efficacy); (c) quality of treatment; (d) curative or non-curative improvement.
RQ4	Google reviews	Direct recommendation given in review Y/N	Not applicable.
RQ5	Google reviews	Type of person making review	(a) patient; (b) family of patient; (c) friend of patient; (d) no relationship to patient; and (e) unspecified.
		Patient cancer stage	(a) stage 1; (b) stage 2; (c) stage 3; (d) stage 4 or terminal cancer.
		Treatment(s) received	Iteratively tallied.
		Amount of money spent	Iteratively tallied.
RQ6	Google reviews	Clinic responses to negative reviews Y/N	Not applicable.
		Positive reviews disputing negative reviews Y/N	Not applicable.

## Results

### Clinic representation of expertise and qualifications

**Fig. 1:**
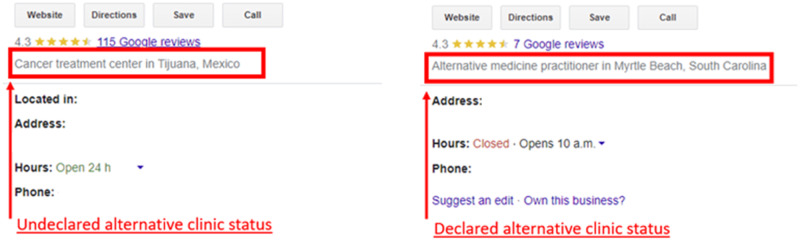
Example of difference in alternative cancer providers Google listing statuses.

Google listings of alternative cancer treatment providers rarely declared that the clinic was an alternative clinic versus a conventional primary cancer treatment provider. Of the 47 clinics, only 6 (12.8%) reported their clinic ‘alternative’, whereas 39 (83.0%) were labeled a cancer/medical clinic or hospital. In 2 (4.3%) cases, clinics were not labeled or had another unrelated label. Figure 1 displays an example of how listings appear.

### Alternative cancer clinics overall ratings

**Fig. 2:**
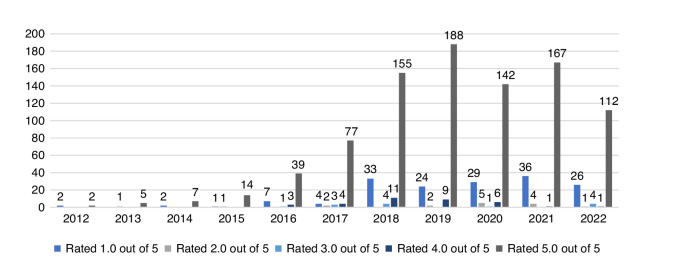
Alternative cancer provider Google review scores by year of reviews.

The clinics were rated highly. On average, clinics received 4.42 stars (median, 4.5; highest score, 5; lowest score, 3.2). Two clinics did not have any ratings at the time of data collection. Clinics had on average 30.7 reviews (median, 19; highest, 127; lowest, 0). Clinic ratings and number of reviews refer to the overall rating information presented on Google business panels and include excluded reviews. Figure 2 provides an overview of included rating scores by year from 2012 to Aug 2022.

### Positive ratings: frequency, source, and stated reasons

943 reviews (83.0%) received 4 or 5 stars. Positive ratings came from patients themselves (*n* = 442), family of patients (*n* = 93), unspecified sources (*n* = 38), a person not related to a patient (*n* = 17), and a friend of the patient (*n* = 7). Positive reviews typically came from people with progressed or late-stage cancer cases. Of the 98 positive reviews where a cancer stage was indicated, 79 (80.6%) came from a person with stage 4 or terminal cancer, 14 (14.3%) stage 3 cancer, 3 (3.1%) stage 2 cancer, and 2 (2.0%) stage 1 cancer.

The most common reason observed for a positive review was the quality of treatment received (*n* = 519, 55.0% of positive reviews) (Table 2). Here, reviews outlined the quality of efficacy-focused cancer treatments, the clinic treatment approach, and provider expertise. Specific reasons given outlining the reasons for a positive review were (1) the treatment avoided the adverse impacts and limitations of conventional cancer care (for example, the harms of chemotherapy or radiation) (*n* = 146); (2) the treatment approach was holistic, whole-body, and integrative (*n* = 83); (3) personalized to the patient (*n* = 69); (4) the treatment used the latest scientific or technological advancements (*n* = 43); and (5) the treatment focused on natural approaches to healing vs. pharmaceutical strategies (*n* = 18).

**Table 2:** Reasons for positive Google reviews on alternative cancer treatment providers by characterization, frequency and example.

Reasons	Characterizations	Frequency	Example
Treatment quality	Treatments offered; non-toxic approaches; avoided limitations of conventional options; provider expertise; holistic; integrative; latest technology; scientific; natural.	519	“Battling stage 4 breast cancer, Doctor’s in America said I would die and Chemo maybe can give me 5–10 years, fast forward six months later the results are PROOF there is an alternative! you must know it is not a death sentence and there ARE other treatments out there.”
Quality of non-intervention care	Communication; attitude; helpfulness; availability; organization; connection; genuineness.	420	“Everyone made me feel very comfortable. The staff was extremely awesome they always took the time to answer my questions and help me with concerns. They made me feel like family.”
Treatment outcomes	Curative, remission achieved, or life meaningfully prolonged	288	“I absolutely recommend [anonymized clinic name]. I went to [clinic name] in August 2021 with stage 4 metastatic thyroid cancer and I am now in remission.”
	Non-curative cancer improvement	78	“It has been an amazing change these past 6 weeks. I have energy, my mental attitude has changed positive, I have hope and look forward to life.”
Facility	Comfortable; conducive to healing; modern; positive aspects of structure and appearance.	62	“The whole place has this healing vibe…..I watched the sunset over the Pacific Ocean most evenings from my room. Everything about this place was therapeutic for me.”

Reviews saw high satisfaction with non-intervention (non-efficacy) care (*n* = 420, 44.5% of positive reviews). Reviews outlined their satisfaction with healthcare workers’ communication, attitude, helpfulness, availability, and organization of services. Quality of care includes cases where reviews indicated they felt their healthcare providers cared for or connected with the patient or their network.

Next, the outcome of the treatment received contributed to high reviews. 33.5% of positive reviews (*n* = 316) documented the services to lead to an improvement. In 288 (30.5%) positive reviews, the treatment allegedly led to cancer being cured, in remission, or the patient’s life meaningfully extended. Among reviews with a declared cancer stage, alleged improvement came most often from persons with stage 4 or terminal cancer (*n* = 68) (Table 3). In 78 (8.3%) positive reviews, treatment allegedly led to cancer symptom improvement or in general functioning.

**Table 3:** Review presenting alternative cancer treatment leading to curative improvement, remission, or meaningful life prolongment and non-curative improvement by patients’ cancer stage by frequency with example.

Improvement Type	Cancer Stage	Frequency	Example
Curative, remission achieved, or life meaningfully prolonged	Stage 1	2	“My brother-in-law started his cancer treatment journey here after being diagnosed with terminal cancer. He is now cancer free from a cancer they said you couldn’t cure.”
	Stage 2	2	
	Stage 3	13	
	Stage 4/Terminal	68	
Non-curative cancer improvement	Stage 1	0	“I saw him deny wheelchair and take walks with me he even did dishes one day and said, ‘I feel good so why not!’“
	Stage 2	1	
	Stage 3	4	
	Stage 4/Terminal	16	

Last, positive reviews commented on the facility quality where they received treatment (*n* = 62; 6.6% of positive reviews). Reviews documented how the facility is comfortable, conducive for healing, modern, and other qualities related to physical structure and appearance.

To encourage others to use the services of a specific alternative cancer clinic, 151 (16.0%) positive reviews contained an action recommendation statement. Reviews prompted and persuaded prospective patients to receive services. For example, phrases such as “do it” and “you need to be here” were found.

### Negative ratings: frequency, source, and reasons

Negative reviews (1- or 2-star ratings) comprised 15.8% (*n* = 180) of the sample. In contrast to positive reviews, negative reviews primarily came from the family of patients (*n* = 60), and very few reviews came from patients themselves (*n* = 17). Reviews also came from unspecified sources (*n* = 41), friends of patients (*n* = 11), and persons unrelated to a patient (*n* = 3). A reason for the change in frequency between the lower number of patient negative reviews compared to patient positive reviews is that many patients with negative reviews were reported to have died following treatment (*n* = 51). Like positive reviews, when cancer stage was declared, most had stage 4 or terminal cases (*n* = 20) or stage 3 cancer (*n* = 3).

37 negative reviews provided information on the amount of money spent on treatment. In total, it was reported that approximately $1,784,645 (average; $48,233.65; median, $25,000) was spent on treatment. However, the figures reported are driven by an outlier review where $500,000 was claimed to be spent on services received. It was also unclear which currencies reviews referred to due to locations of both patients and clinics. The number reported here underreports true spending because few reviews reported the full amount spent on treatment and it is impossible to ascertain spending based solely on review content.

The most prevalent reason for a negative review was the allegation that alternative cancer clinics financially exploit patients with ineffective treatment (*n* = 98, 54.4% of negative reviews) (Table 4). Here, reviews alleged clinics were in pursuit of money, not healing; employed false hope to attract patients; were scams; and the treatments offered do not work there. In 21 (11.7%) negative reviews, it was stated the clinics communicated that they could cure or prolong the life of the patient receiving services. In 5 cases, reviews stated the clinics gave a percentage chance of success ranging from 70 to 90%.

**Table 4:** Reasons for negative Google reviews on alternative cancer treatment providers by characterization, frequency and example.

Reasons	Characterizations	Frequency	Example
Perception clinic in pursuit of money, not healing	False hope; scam; ineffective treatments; financial exploitation; services to people who can’t be helped.	98	“My friend had stage 3 colon cancer; she did the 30 day stay for $30 k. She died weeks later after coming back home. My opinion, this place is a scam job with scam artist.”
Treatment worsened Patient’s condition or cancer progressed	Weakened patient; specific injuries; complications; opted out conventional treatment and disease progressed; death or near death.	72	“Not only was their treatment useless, it made matters worse. They essentially let her starve to death and chastised her for seeking help at the local hospital when her symptoms became unbearable.”
Quality of non-intervention care	Communication issues; rudeness; cold; staff availability; poor training; disorganization; related issues.	41	“When my wife was there they didn’t even have a registered nurse available during the night!”
Misrepresented impact of received treatment on cancer	False, unsubstantiated claims of patients’ treatment impact; fabricated or exaggerated testimonials	23	“Classic alternative medicine type stuff, mostly a scam. Tests can come back negative and show no evidence of disease, etc., but get a CT scan back in the U.S. and it shows spreading of cancer.”
Facility	Underequipped; dirty; unsafe; dated.	8	“They performed port surgeries in an extremely unhygienic environment.”
Time away from loved one’s while receiving treatment	Time lost for family, friends, other items due to receiving treatment	7	“My mom is home now in the final stage of her cancer. She spent a precious month in Arizona away from her home and family.”
Difficulty accessing treatment	Issues related to scheduling, initial consultations	3	“They do not answer the phone and their website does not exist.”

Next, negative reviews were posted because the treatment worsened the patients’ conditions or that cancer progressed following treatment (*n* = 72, 40.0% of negative reviews). This included cases where the patient or someone in their network felt weaker, had specific injuries, or had care-related complications. The most concerning cases included cases where cancer progressed after opting out of conventional care in favor of alternative cancer treatment. Following treatment, 51 persons were reported to have died, of which five were reported to have done so in the custody of the alternative cancer provider.

Negative reviews were given for the quality of non-intervention care in 41 (22.8%) cases. Complaints recorded included issues related to healthcare workers’ communication (language barriers), attitude, availability of healthcare workers, quality of care workers (training), understaffing, and organization of services.

In 23 (12.8%) negative reviews alternative cancer clinics are said to have misrepresented to a patient, their family, or another representative about the impact of received treatment on cancer. Accusations include the clinic telling a patient either: (1) the treatment had cured them; (2) they were in remission; (3) the size of their tumor was decreasing; or (4) the treatment was working. The reviews detailed situations where patients and their loved ones received positive news at the clinic, but upon travel home or independent testing, they were told the opposite, where their cancer had worsened. For example, a review for a clinic in Arizona states: “we were told her CT scan showed a “37% reduction of the cancer,” only to have her oncologist from home confirm that her cancer was in fact WORSE & SPREADING.” A review of a Tijuana clinic, similarly, reads: “My husband went there twice. The second time they told him he was cancer free and sent him home. He was not cancer free. It had even spread to his liver and bones.” In some reviews, it was alleged the misrepresentation of the treatment’s impact on cancer was a strategy to continue expensive treatment with the clinic. For example, a review of a Tijuana clinic states: “they would tell her [that her] tumour was going down and getting better and US doctors looked at her crazy and said her cancer spread… This hospital lies to you just to keep you going and getting your money.” Supplementary Information File 2 provides illustrative examples of situations where treatment outcomes from alternative cancer clinics are alleged to have been mispresented.

Less commonly reported reasons for negative reviews included the quality of the facility (*n* = 8, 4.4%), time away from loved one’s while receiving treatment (*n* = 7, 3.9%), or difficulty accessing treatment (*n* = 4, 2.2%). The characterization and an example of each code is given in Table 3.

### Reputation management

Alternative cancer clinics responded to 35 negative reviews to dispute reviews detailing improper conduct. Clinics often sought to refute that their interventions did not work and that their providers were not qualified (*n* = 16). In response to patients’ condition worsening or dying, clinics stated that patients die due to aggressive or terminal cancers even when helpful treatment is offered (*n* = 8). In more aggressive rebuttals, clinics asserted that (1) negative reviews were fraudulent (*n* = 7); (2) that they do not make promises about treatment efficacy, promises of recovery, and that they only take patients they can help (*n* = 7); (3) it was the result of patients actions, not their own, that their situation not did not improve (*n* = 6); and (4) clinics do not lie about improvement or fake testimonials (*n* = 4). In softer rebuttals, clinics: (1) offered sympathy and prayers (*n* = 13) or (2) shared their institution’s history (*n* = 6). Sometimes, clinics apologized and made offers to correct the situation (*n* = 6). Rebuttals and responses are summarized in Supplemental Information File 3.

Among positive reviews, we found evidence certain Google reviews challenged negative reviews (*n* = 21). Here, reviews referenced the negative reviews and provided a rebuttal. For example, a review for [anonymized clinic name] hospital states: “I saw a post here talking about people being promised a cure. That is a lie. I have NEVER heard ANY of the doctors promise anybody a cure. That is completely asinine and really ticks me off that someone would lie like that in the comments!” It is not possible to state whether the reviewer had an affiliation with the clinic.

## Discussion

Our findings suggest alternative cancer clinic Google listings and reviews create a favorable online impression to prospective patients. In nearly all cases, from search results, alternative cancer clinics are labeled as specialty primary cancer options. Only a few clinics are marked as an ‘alternative’ option. Clinics have many reviews, nearly all of which are rated highly. Positive reviews state treatments can cure cancer or prolong life, even in previously considered terminal prognoses. Positive reviews also undermine evidence-based cancer treatments such as chemotherapy in favor of alternative and holistic approaches. Negative reviews are less frequent but contain concerning allegations of financial exploitation and misrepresentation of treatment impacts.

The favorable Google search and review results of alternative cancer clinics contribute to harmful online ecosystems that provide legitimacy to clinics and false hope to very ill persons [7]. Cancer patients with late-stage or terminal cancers, who have been advised that there are no further treatment options, may reasonably search for solutions elsewhere [15]. Patients, though, are very unlikely to be qualified to evaluate the effectiveness of cancer treatments or the research supporting them [36], and patients also experience confirmation or optimism biases [37], where ‘red flags’ may be ignored in pursuit of hope. Unfortunately, the pursuit of false hope makes patients and their families vulnerable to financial exploitation [38] through exposure to harmful information and services from uncredible sources. This can take the form of social media misinformation [36, 39, 40], exploitive ads [8], support groups [6], crowdfunding campaigns [3, 41, 42], forums, search results [43], or websites [44]. Alternative cancer clinics’ use of Google listings and reviews represents one source of harmful information. Clinics can shape their reputation by labeling themselves ‘cancer treatment centers’ or ‘cancer specialists’ in their Google business profiles. For many clinics, this offers an affordance of legitimacy. It also affords a unique, unchallenged, brand-shaping opportunity to prospective patients, who likely see their Google description as their first point of contact upon searching on Google, positioning themselves as legitimate healthcare providers.

Testimonials from Google reviews, although unverified, are compelling narratives [45] to potential patients but are an ineffective indicator of treatment quality and impact on outcomes. The veracity of claims found in the reviews in our sample, which frequently provide narratives of alternative cancer treatments leading to a cure or remission, should be interpreted cautiously. The prevalence of fraudulent reviews are high across Google reviews for many industries. A thriving, largely unregulated, fake reviews market distorts ratings on Google and other rating sites [46]. Medical providers, such as physicians, are reported to purchase fake Google reviews to attract patients [30, 47]. This is largely effective. Patients prefer online physician reviews compared to government ratings [48] and a 2020 survey suggests over 71% use online reviews to assess options for a new healthcare provider. Fabricating Google reviews for medical purposes can result in worse care than expected [15]. However, more severe cases, such as alternative cancer clinics, can lead to devastating treatment decisions and outcomes. Persons with cancer may opt out of evidence-based treatment, spend thousands on ineffective treatment, reduce their life expectancy, and spend critical time away from loved one’s while receiving unnecessary alternative treatment. The impact and burden this has on the mental health and wellbeing of bereaved family members remains under-researched.

Google has some but not unlimited ability to meaningfully restrict businesses’ online presence on its site. This may lead to alternative cancer clinics potentially exploiting prospective clinic patients. Google business profiles are created in two ways: (1) businesses provide information, become verified, and an account is made; or (2) Google collects online data and creates a business profile for them, which the business itself can later claim. In descriptions, businesses may not “mislead” users with “inaccurate” or “deceptive information [49].” Google allows businesses online to provide a description of their services (written by businesses themselves) and categorize their business (from pre-existing Google set options). Categorizations, which “help… customers find accurate, specific results for services they’re interested [in]” are selected and updated by businesses themselves [49]. Categories are used to provide relevant search results for Google search users, but this is where alternative cancer clinics can inaccurately report their services. There are thousands of business categories, including options such as ‘alternative medicine practitioner,’ ‘naturopathic practitioner,’ and ‘holistic medicine practitioner,’ among other options. In our study, however, nearly all clinics label themselves a ‘cancer treatment center’ and similar categories (for example, hospitals). Google does not suspend business profiles so long as they do not include fraudulent or illegal content, but it is unclear if and how Google enforces these definitions. This could have contributed to the phenomenon of alternative cancer clinics inaccurately representing themselves to appear in inappropriate search results.

Similarly, the limited ability of Google to effectively moderate the accuracy or veracity of Google reviews puts cancer patients at risk of inaccurate reviews from alternative cancer clinics. Google prohibits ‘deceptive content’, which includes fake engagement (‘content that does not represent a genuine experience’), misinformation (‘false or inaccurate information that may cause significant harm to individuals, businesses, or society’), or misrepresentation (‘misleading representations or omissions to gain improper benefits’) [50]. To enforce guidelines, Google relies upon user reporting, its enforcement teams, and artificial intelligence detection. When violations are found, Google removes the offending content. However, the problem arises that it is not feasible for Google to adequately monitor the informational quality of the tens of millions of reviews it hosts monthly. Concerns surrounding the accuracy of fake reviews have spurred regulatory agencies, such as the Federal Trade Commission, to propose initiatives and heavy penalties to curb fake reviews [51]. Google has expressed willingness to assist regulatory agencies combat fake reviews and misleading endorsements [52]. However, this context leads to significant questions about the accuracy of medical review content, including alternative treatment reviews. In our study, we cannot assess the degree of review truthfulness. However, we can state unequivocally that effective safeguards to ensure truthful, accurate medical reviews are absent, and thus any alternative cancer clinic Google review should not be used for medical decision-making.

We do not make any assumption that Google purposefully allows or means for this conduct to occur, but rather, its systems are actively abused with limited, potentially negligent [53], oversight. Since Google is under no obligation to remove fake reviews, we do, however, question their commitment to change systems and we call for immediate interventions. To protect vulnerable groups, such as persons with cancer, we encourage Google to provide better safeguards and offer several recommendations on this. First, alternative cancer clinics should not be able to label themselves as conventional cancer providers. They should be identified clearly as an alternative provider if they have a business presence on Google. Second, warnings should be placed on questionable medical advertisers with linkages to qualified sources of information, such as the American Cancer Society or conventional health provider organizations, such as the American Medical Association. Third, Google should not recognize alternative health professions, such as naturopaths or chiropractors, with the same status as conventional health provider groups, such as nurses, physicians, physiotherapists, and occupational therapists, when making content decisions [54, 55]. Fourth, Google should warn people reading medical clinic reviews that reviews are not substitutes for medical advice and that Google cannot confirm the veracity of claims. Fifth, Google must provide targeted enforcement to audit the truthfulness of reviews for health-related businesses. Last, we encourage Google to cease other business activities with alternative cancer clinics’ use of their products, such as demonetizing their YouTube accounts, prohibiting their use of Google ads, and reducing search result priority.

Future research is needed to explore how online systems enable the exploitation of cancer patients. Areas of particular concern include investigating how alternative cancer clinics use social media platforms to market their services. Research should focus on the normative factors that enable clinics to attract prospective patients versus solely the clinics’ online activities. This includes examining the policies of social media platforms [56] and other online sources that are used to promote alternative cancer clinics. Survey and qualitative research are also needed to determine if alternative cancer clinic marketing reaches prospective patients and, if so, how it is interpreted.

Our study faces several limitations. First, we sampled prominent alternative cancer providers that target English-speaking clients. Second, we cannot confirm the truthfulness of Google review content. It may be that reviews offer degrees of truth or inaccuracies. Nonetheless, we emphasize that frequent narratives of persons with late-stage cancer being cured or in remission cause false hope and are, therefore, harmful. Last, some clinics treat other diseases and ailments than cancer, but their reviews did not provide a text description of the reason for their rating score. In these cases, the review score is included in the overall score reported, but we cannot confirm the rating specifically refers to cancer services.

### Supplementary information

Standards for Reporting Qualitative Research (SRQR)

Supplementary Information File 2

Supplementary Information File 3

## Data Availability

The datasets generated during and/or analysed during the current study are available from the corresponding author on reasonable request.
