# Supplementary material for: Alternative cancer clinics’ use of Google listings and reviews to mislead potential patients
Source: BJC Rep. 2024 Aug 6;2:55. doi: 10.1038/s44276-024-00071-9 (PMC11303243; doi:10.1038/s44276-024-00071-9)
Supplement: Supplementary file 2 — Supplementary Information File 2 [file 44276_2024_71_MOESM2_ESM.docx]

Supplementary Information File 2. Alternative cancer clinics alleged to misrepresent the impact of their cancer treatment by clinic name, frequency, and example

| Clinic Name | Frequency | Example |
| --- | --- | --- |
| [Anonymized Clinic] | 4 | "I went through their program and **they gave me their word that the tumors have shrunk. I have no proof** however. I asked for the ultrasound images but all they gave me was a corrupted disk that won't read…" |
| [Anonymized Clinic] | 4 | "My husband went there twice. **The second time they told him he was cancer free and sent him home. He was not cancer free.** It had even spread to his liver and bones. We learned that after his first scan after arriving back to the U.S. They even had him make a little tutorial for their Facebook page saying he was leaving cancer free." |
| [Anonymized Clinic] | 3 | "When her health struggled, **we were told that her treatment was "working" & that it was "normal." After 3 months of this roller coaster****, we were told her CT scan showed a "37% reduction of the cancer**," only to have her oncologist from home confirm that her cancer was in fact WORSE & SPREADING." |
| [Anonymized Clinic] | 2 | "All they do is rob people of their life savings, and they don't have any cures, just false hope and rob desperate families of their life savings. **Most of the reviews on here are done by people that work at the clinic**, FAKE HOPE DO NOT BE FOOLED." |
| [Anonymized Clinic] | 2 | "**My sister cancer spread and they would tell her tumor was going down and getting better** and U.S. doctors looked at her crazy and said her cancer spread and she had more than one tumor. This hospital lies to you just to keep you going and getting your money." |
| [Anonymized Clinic] | 1 | "Well after months and months and months of treatments and my health getting worse and worse, **they finally said oh you're getting better. I said but my markers are getting higher** they said well we don't go off the markers no more! Seemed kinda odd when that's all they went off since the start and I paid to get blood drawn every week to check my marker levels." |
| [Anonymized Clinic] | 1 | "Not only not working but he was significant worse since the start of treatment. Grandpa calls [anonymized clinic name] and of course **they want him to stay on it because it's working "10" %!!!!!! No tests on the cancer or medication from afar to even take a random guess it's working 10%!"** |
| [Anonymized Clinic] | 2 | "**2 weeks at [anonymized clinic name] they said from their testing his cancer was cured and dead.** He flies home thinking he was cured, 2 weeks later he was in hospital coming in and out of consciousness, the scans showed that he was never cured of his cancer, it in fact metastasized everywhere and he was now dying, all organs were shutting down." |
| [Anonymized Clinic] | 1 | "Several times she was treated in this terrible clinic, always with the words: **come back we are on the right track.** In the end, [anonymized clinic name] managed a 10 cm metastasis in her liver in one year for about 60,000€" |
| [Anonymized Clinic] | 1 | "**Their post treatment evaluation showed insignificant improvement of the disease** however the diagnostic tools that were used were NOT the appropriate ones for my type of cancer and they released me with a recommended further procedure for therapy at home that consequently led to the dramatic progression of the disease." |
| [Anonymized Clinic] | 1 | "He kept telling **her you're doing good you're doing good** and now look what happened" |
| [Anonymized Clinic] | 1 | "From the group of 7 co patients I kept contact after leaving the clinic almost 2 years ago I am the only one still alive. (I am on conventional chemo). **Several were told to be cancer free on completion of the [anonymized clinic name] treatment but didn't last long. Lots of false hope**!!Note that all the wonderful testimonials are done while patients are still hyped up by the impressive treatment at the clinic. There are none conducted with patients months or years after leaving the clinic!" |
