# Supplementary material for: Alternative cancer clinics’ use of Google listings and reviews to mislead potential patients
Source: BJC Rep. 2024 Aug 6;2:55. doi: 10.1038/s44276-024-00071-9 (PMC11303243; doi:10.1038/s44276-024-00071-9)
Supplement: Supplementary file 3 — Supplementary Information File 3 [file 44276_2024_71_MOESM3_ESM.docx]

Supplemental Information File 3. Alternative cancer treatment providers whose rebuttals comment on negative reviews by frequency and example

| **Response** | **Frequency** | **Example** |
| --- | --- | --- |
| Clinicians are qualified/treatments are evidence-based, scientific, and work | 16 | "The qualifications of the chief physician are of course substantiated and verifiable, in addition to publications in specialist journals and lectures, regular lectures at the Universities of Seville and Giessen, supervision of doctoral theses in the field of oncological and complementary medicine." |
| Sympathy/thoughts/prayers | 13 | "Again, we are sorry to hear of your family member's passing and hope you will allow us to pray for you and your family." |
| Sometimes patients die due to unrelenting cancer even with treatments that may help | 8 | "Anytime a patient passes we wish we could have done more to help. Our medical team provided the best care possible. Unfortunately, not all outcomes are favorable." |
| Do not make promises about treatment efficacy or success rates and only take patients they can help | 7 | "Regardless of the stage of an illness, we never make a promise of salvation. That would be dubious and would certainly not have resulted in our clinic having existed for more than 35 years." |
| Review is Fraudulent | 7 | "Google has removed these reviews several times and has also encouraged us to report and ban these users; however, we have come to the conclusion that it is in everyone's best interest that we respond instead." |
| Commenting on history of clinic | 6 | "[anonymized clinic name] was founded 20 years ago and has been treating patients with all different types and sizes of tumors as you can read in our Patient Testimonials on our website." |
| Apologizing and/or wanting to make client negative experience right | 6 | "… we have seen your review and since we do not know how we may have disappointed you, we want to ensure that if there is anything we can do to help you or a loved one, we are available by telephone and email." |
| Patients' actions, not clinic | 6 | "We know how to save lives of cancer patients, but only if they take our advice. Staff has confirmed several points of dispute between us and the patient." |
| Do not lie about improvement or fake testimonials | 4 | "… our reports showed improvements but never did we lied to you or send you home intoxicated… Our testimonials are as real as they can get, 100% of the testimonials and people you see in our social media are patients or companions." |
